# Brachial Plexus Injury Secondary to Spontaneous Upper Limb Haematoma

**DOI:** 10.7759/cureus.55693

**Published:** 2024-03-06

**Authors:** Natasha Aghtarafi, Natalia Makhdoom, Ali Arnaout, Kai Yuen Wong

**Affiliations:** 1 Radiology, East Suffolk and North Essex NHS Foundation Trust, Ipswich, GBR; 2 General Surgery, Birmingham Women's and Children's NHS Foundation Trust, Birmingham, GBR; 3 Plastic and Reconstructive Surgery, Cambridge University Hospitals NHS Foundation Trust, Cambridge, GBR

**Keywords:** brachial neuropraxia, spontaneous hemorrhage, painful neuropathy, brachial plexus injury, therapeutic anticoagulation, arm compartment syndrome

## Abstract

Spontaneous upper limb muscle haematomas are rare clinical phenomenons, which often go under- or misdiagnosed. They can present management challenges in the context of anticoagulant therapy, especially in the presence of other medical conditions. We present the case of a 52-year-old male with an initially missed presentation of a spontaneous muscle haematoma that progressed and re-presented to the emergency department (ED) with signs of mixed upper limb neuropathy requiring surgical evacuation and an emergency fasciotomy. This case highlights the importance of prompt diagnosis and intervention. While brachial plexus injuries from haematoma compression are uncommon, in our case, we discuss the need for surgical intervention to relieve pressure and optimise patient outcomes when clinically concerned about compartment syndrome or progressive neuropathy.

## Introduction

Spontaneous muscle haematomas are characterised by the unprovoked extravasation of blood within muscle groups. They are commonly reported in patients on anticoagulation in specific anatomical regions, such as the iliopsoas, rectus sheath and gluteal muscles [1,2]. These occurrences, often underestimated or misdiagnosed, gain particular significance in the context of anticoagulant therapy and can have serious consequences if not recognised or treated in a timely manner [2]. 

Anticoagulant therapy, in particular warfarin, increases susceptibility to spontaneous bleeding. The reported incidence of these events in patients receiving warfarin is up to 7.2% [3], particularly when the international normalised ratio (INR) exceeds therapeutic thresholds. Coagulation-induced haemorrhage has an increased annual mortality rate of approximately 0.65% [4].

## Case presentation

A 52-year-old male presented to the emergency department (ED) with a two-day history of spontaneous swelling, redness and discomfort in his upper left arm. Given the absence of trauma, he was diagnosed with upper limb cellulitis and discharged home with a course of oral antibiotics.

**Figure 1:**
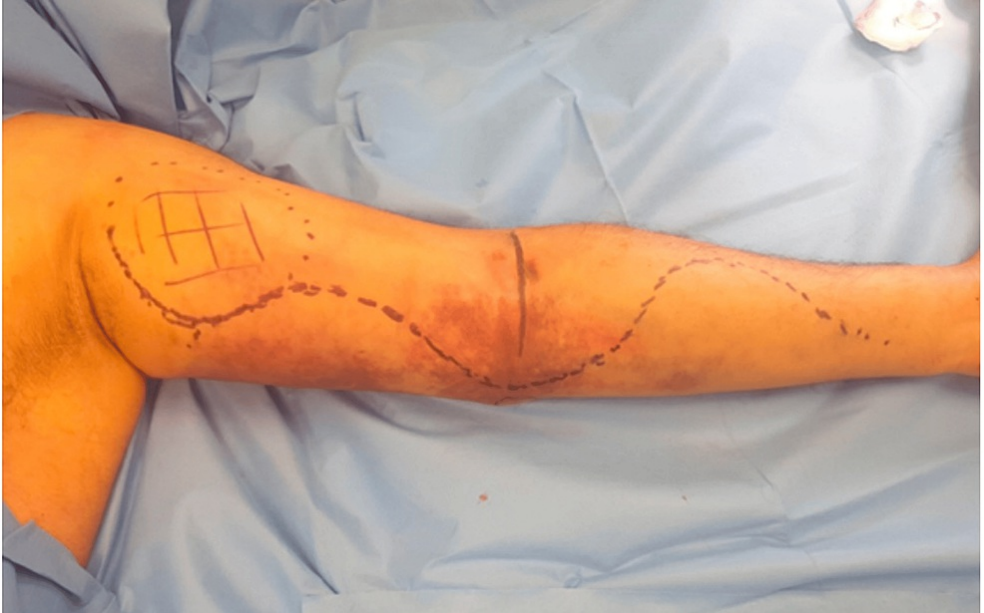
Preoperative appearance of the left arm and forearm. Left arm and forearm preoperatively, demonstrating the extent of the bruising and swelling.

On the following day, he returned with a significant progression of his symptoms and worsening pain. A physical examination revealed swelling and ecchymosis in his left upper arm (Figure 1). He had signs of mixed upper limb neuropathy, primarily affecting the lower trunk of the brachial plexus (Table 1). His arm was uncomfortable at rest, with severe pain during passive movements.

**Table 1:** Muscle power during admission, four and 18 months post discharge.

Medical Research Council (MRC) Muscle Scale 1943 (Grades 0-5)			
	During admission	4 months post admission	18 months post admission
Biceps/brachialis C5/6	1	3	5
Brachioradialis C5/6	1	3	5
Flexor carpi radialis C7	2	3	5
Extensor digitorium C7	1	1	4
Extensor indicis proprius C7	1	1	4
Extensor pollicis longus C7	1	1	1
Extensor digiti minimi C8	1	1	4
Flexor digitorum superficialis /profundus C8	3	4	5
Flexor pollicis longus C8	3	4	5
Abductor pollicis longus T1	3	4	5
Adductor pollicis longus T1	3	4	5
Intrinsic muscles T1	1	1	4

**Figure 2:**
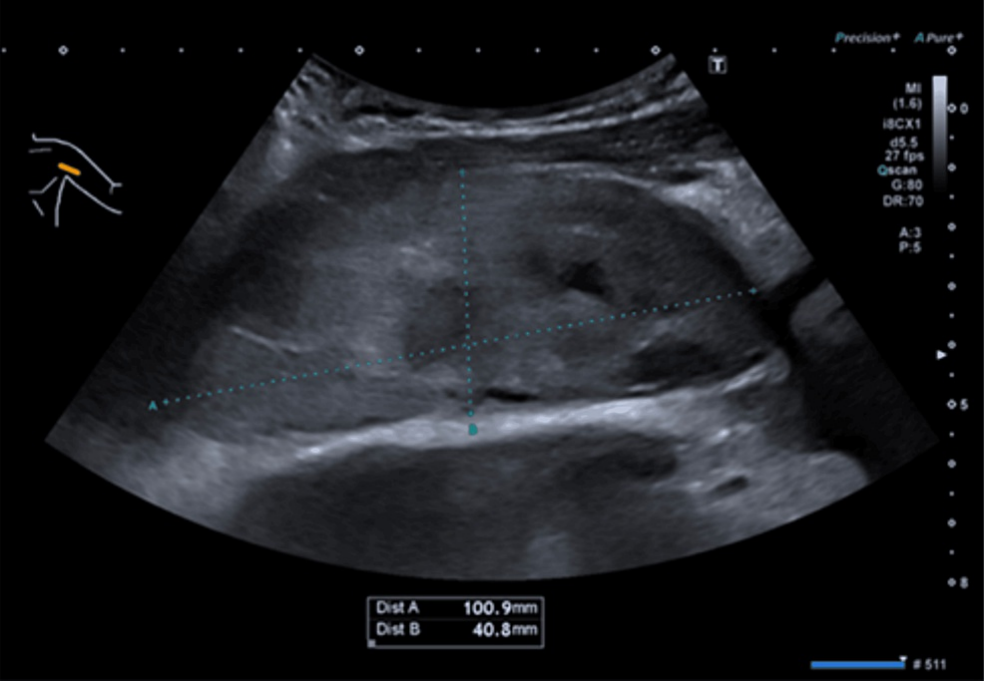
Ultrasound scan of the haematoma in the left axilla. Ultrasound scan showing a large well-defined area of mixed echogenicity measuring 10 x 5 cm (longitudinal x AP). In keeping with an intramuscular haematoma of the left axilla.

His past medical history included a metallic aortic valve, necessitating warfarin therapy with a target INR of 2.5. During his second presentation to the ED, the INR was 5.3, exceeding the therapeutic threshold for a metallic valve and posing a bleeding risk. This raised concern for a potential hematoma, especially as the patient denied any precipitating events like trauma. An urgent upper limb ultrasound revealed a sizable 10 x 5 cm intramuscular and subfascial hematoma in the left axilla (Figure 2).

Given the symptom progression and signs of compressive neuropathy, warfarin was discontinued, and the INR was corrected with intravenous vitamin K based on advice from the local haematology team. Subsequently, the INR decreased from 5.3 to 1.4. The patient was bridged with intravenous heparin to mitigate the thrombotic risk associated with the metallic valve replacement. 

**Figure 3:**
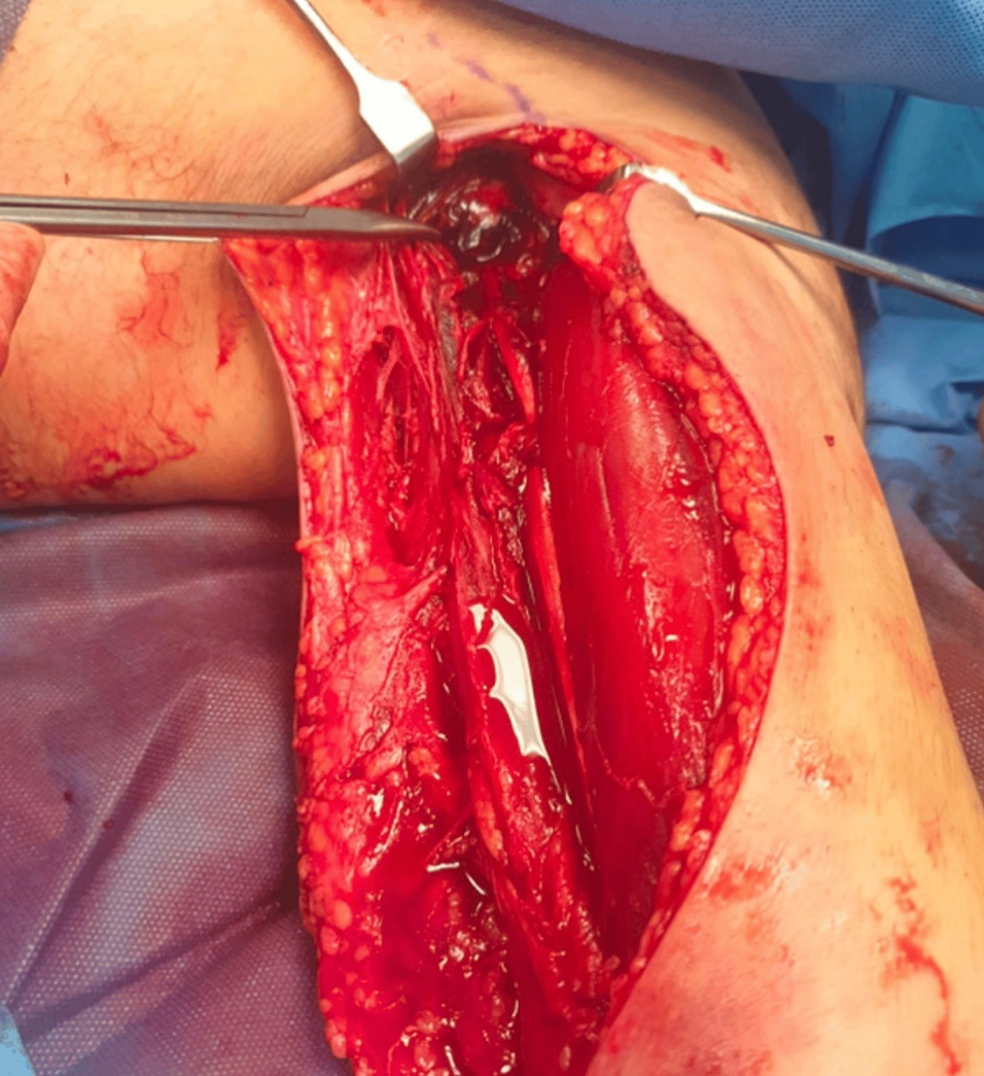
Post-evacuation intraoperative photo, demonstrating residual haemorrhage in the axilla.

Once the INR had reduced to a safe threshold, the patient was taken to the operating theatre for an emergency fasciotomy and hematoma evacuation due to the clinical concern of compartment syndrome. Intraoperatively, a large intramuscular hematoma with a volume of 200 ml compressing the coracobrachialis muscle and extending into the left axilla was found (Figure 3). Exploratory fasciotomies of the arm and forearm confirmed soft compartments and healthy muscle.

Four months postoperatively, there was partial recovery of the brachioradialis muscle, but the musculocutaneous, ulnar, and median nerves showed improvement, as demonstrated in Table 1. 

Two years postoperatively, the patient experienced a good recovery of the ulnar and musculocutaneous nerves and a further improvement in the radial nerve, achieving full power in the triceps and wrist extensors. However, there was poor recovery of the extensor pollicis longus (EPL) muscle with ongoing 1/5 power (Table 1).

Tendon transfer was offered as a treatment, but the patient declined and is continuing physiotherapy with the hand therapy team. 

## Discussion

The prevalence of spontaneous muscle haematomas has increased with an increase in the use of anticoagulation, especially in the elderly [5]. Clinical symptoms can vary widely depending on the location and volume of the haematoma, with patients presenting with pain, swelling or an acute drop in haemoglobin [6,7].

The management of haemorrhage in an anticoagulated patient can prove to be challenging if there are certain underlying medical conditions, such as atrial fibrillation or implanted heart valves [8]. In such cases, it is important that there is a balance between the risks and benefits of the treatment.

In this case, the patient was anticoagulated with warfarin for a metallic aortic value, for which the therapeutic range is between 2.5 and 3.0. An INR of 0.9-1.2 is considered normal [8,9]. Phytmenadione, more commonly known as vitamin K, synthesises vitamin K-independent clotting factors and is widely used for the reversal of warfarin-induced bleeding or INR <10. It is given either orally or intravenously, and its effects begin one to two hours after administration [10]. 

Brachial plexus neuropathy stemming from spontaneous muscle haematoma is uncommon, with brachial plexus injuries more commonly occurring due to traumatic injuries, malignancies or iatrogenic causes [11]. Motorbike-related accidents are the most common cause of traumatic brachial plexus injuries and can result in severe neurological complications, leading to upper limb functional impairment [12,13].

Frangides and Kounis (1992) published a three-case series of brachial plexus palsy secondary to anticoagulation-induced haematoma. All three cases were over the age of 60 with diminished reflexes. They were managed conservatively with anticoagulation reversal using vitamin K and physiotherapy. Despite presenting with severe upper extremity neurologic deficits, none of the patients required surgical management, and all showed good recovery, with one patient experiencing a prolonged neurological deficit. In usual practice, a prolonged deficit is considered longer than 18 months [14]. 

Compressive neuropathies are generally treated conservatively, and nerve recovery is variable, with most patients experiencing full recovery of function. However, some patients may have incomplete or delayed recovery due to axonal loss [15,16]. However, the degree and speed of recovery can be variable, and some patients may experience residual deficits. [11,12].

In our case, at 18 months post-haematoma, the patient demonstrated full recovery of the flexors and most of the extensor muscles, with the exception of EPL, which remained at 1/5 power (Table 1). EPL is a deep muscle of the posterior forearm and is innervated by a motor branch of the posterior interosseous nerve (PIN) [17]. The radial nerve is superficial and vulnerable to compression, particularly at the axilla and radial groove [18]. While lower extremity neuropathy can be attributed to upper nerve lesions [18], the partial recovery of other extensor muscles innervated by the PIN may indicate that the wrist extensors underwent longer compression than the muscles that demonstrated full recovery.

In cases where brachial plexus injuries occur, such as the one presented here, a multidisciplinary approach is often necessary for optimal patient care. Physiotherapy and rehabilitation play a crucial role in helping patients regain function and mobility. Regular follow-up and monitoring are essential to track progress and address ongoing issues.

## Conclusions

Spontaneous upper limb muscle haematomas in anticoagulated patients are rare but significant clinical entities that demand careful consideration and timely intervention. Managing spontaneous muscle haematomas in patients receiving anticoagulant therapy poses a unique challenge to clinicians, with careful consideration of the risks versus benefits.

While most cases can be managed conservatively, our case demonstrates the importance of timely surgical intervention in the presence of progressive compressive neuropathy. A comprehensive, multidisciplinary approach that includes haematologists, surgeons, and rehabilitation specialists is essential for achieving the best possible outcomes for these patients.

## References

[1] Pode D, Caine M (1992). Spontaneous retroperitoneal hemorrhage. J Urol.

[2] Dohan A, Darnige L, Sapoval M, Pellerin O (2015). Spontaneous soft tissue hematomas. Diagn Interv Imaging.

[3] Xu Y, Schulman S, Dowlatshahi D (2017). Direct oral anticoagulant- or warfarin-related major bleeding: characteristics, reversal strategies, and outcomes from a multicenter observational study. Chest.

[4] Zidane M, Schram MT, Planken EW, Molendijk WH, Rosendaal FR, van der Meer FJ, Huisman MV (2000). Frequency of major hemorrhage in patients treated with unfractionated intravenous heparin for deep venous thrombosis or pulmonary embolism: a study in routine clinical practice. Arch Intern Med.

[5] Sasson Z, Mangat I, Peckham KA (1996). Spontaneous iliopsoas hematoma in patients with unstable coronary syndromes receiving intravenous heparin in therapeutic doses. Can J Cardiol.

[6] Kwon OY, Lee KR, Kim SW (2009). Spontaneous iliopsoas muscle haematoma. Emerg Med J.

[7] Fernandes C, Pereira P, Rodrigues M (2015). Spontaneous iliopsoas muscle haematoma as a complication of anticoagulation in acute cerebral venous thrombosis: to stop or not to stop (the anticoagulation)?. BMJ Case Rep.

[8] Kaneko T, Aranki SF (2013). Anticoagulation for prosthetic valves. Thrombosis.

[9] Levine M, Goldstein JN (2014). Bleeding complications of targeted oral anticoagulants: what is the risk?. Hematology Am Soc Hematol Educ Program.

[10] Yee J, Kaide CG (2019). Emergency reversal of anticoagulation. West J Emerg Med.

[11] Khadilkar SV, Khade SS (2013). Brachial plexopathy. Ann Indian Acad Neurol.

[12] Midha R (1997). Epidemiology of brachial plexus injuries in a multitrauma population. Neurosurgery.

[13] Yoshikawa T, Hayashi N, Yamamoto S (2006). Brachial plexus injury: clinical manifestations, conventional imaging findings, and the latest imaging techniques. Radiographics.

[14] Frangides C, Kounis NG (1992). Anticoagulant-induced shoulder hematoma producing brachial plexus neuropathy--case reports. Angiology.

[15] Ferrante MA (2004). Brachial plexopathies: classification, causes, and consequences. Muscle Nerve.

[16] Sharp O, Wong KY, Stephens P (2017). Backpack palsy with Horner's syndrome. BMJ Case Rep.

[17] Moore K, Dalley A, Agur A (2014). Clinically oriented anatomy. Clinical Anatomy.

[18] Silver S, Ledford C, Vogel K, Arnold J (2021). Peripheral nerve entrapment and injury in the upper extremity. Am Fam Physician.

